# Sap Transporter Mediated Import and Subsequent Degradation of Antimicrobial Peptides in *Haemophilus*

**DOI:** 10.1371/journal.ppat.1002360

**Published:** 2011-11-03

**Authors:** Catherine L. Shelton, Forrest K. Raffel, Wandy L. Beatty, Sara M. Johnson, Kevin M. Mason

**Affiliations:** 1 The Research Institute at Nationwide Children's Hospital, Center for Microbial Pathogenesis, Columbus, Ohio, United States of America; 2 Washington University School of Medicine, St. Louis, Missouri, United States of America; 3 The Ohio State University College of Medicine, Department of Pediatrics, Columbus, Ohio, United States of America; Northwestern University Feinberg School of Medicine, United States of America

## Abstract

Antimicrobial peptides (AMPs) contribute to host innate immune defense and are a critical component to control bacterial infection. Nontypeable *Haemophilus influenzae* (NTHI) is a commensal inhabitant of the human nasopharyngeal mucosa, yet is commonly associated with opportunistic infections of the upper and lower respiratory tracts. An important aspect of NTHI virulence is the ability to avert bactericidal effects of host-derived antimicrobial peptides (AMPs). The Sap (sensitivity to antimicrobial peptides) ABC transporter equips NTHI to resist AMPs, although the mechanism of this resistance has remained undefined. We previously determined that the periplasmic binding protein SapA bound AMPs and was required for NTHI virulence in vivo. We now demonstrate, by antibody-mediated neutralization of AMP in vivo, that SapA functions to directly counter AMP lethality during NTHI infection. We hypothesized that SapA would deliver AMPs to the Sap inner membrane complex for transport into the bacterial cytoplasm. We observed that AMPs localize to the bacterial cytoplasm of the parental NTHI strain and were susceptible to cytoplasmic peptidase activity. In striking contrast, AMPs accumulated in the periplasm of bacteria lacking a functional Sap permease complex. These data support a mechanism of Sap mediated import of AMPs, a novel strategy to reduce periplasmic and inner membrane accumulation of these host defense peptides.

## Author Summary

The opportunistic pathogen *Haemophilus influenzae* is a normal inhabitant of the human nasopharynx, and is commonly implicated in respiratory tract infections, particularly of the middle ear (otitis media), sinuses, and lung (pneumonia, chronic obstructive pulmonary disease and cystic fibrosis). We have identified a multifunctional bacterial uptake system that is required for critical mechanisms of bacterial survival in the host. This Sap transporter system recognizes and transports host immune defense molecules and is involved in uptake of an iron-containing nutrient (heme) that is host-limited, yet required for bacterial growth and survival. We propose that bacteria utilize this, and likely other similar transport systems, for numerous functions that are important for bacterial survival in the host, including host immune evasion and metabolism. Our findings significantly advance our understanding of how single bacterial protein systems co-operate and coordinate multiple functions to equip bacteria to survive and cause disease in the hostile host environment. Our long-range goal is to block this uptake system thereby starving the bacterium of essential nutrients and also promoting clearance by the host immune response. Removal of this important bacterial survival mechanism will thwart the ability for *Haemophilus* to survive as a pathogen and thus decrease the incidence of disease development.

## Introduction

Host-derived antimicrobial peptides (AMPs) are typically amphipathic, cationic innate immune defense molecules that target bacterial membranes, disrupt transmembrane potential and trigger cytoplasmic leakage resulting in bacterial cell death [1], [2]. Defensins (α- and β-) and cathelicidin (hCAP-18/LL37) molecules are primarily abundant in neutrophils (α-defensins and cathelicidin), respiratory epithelium (β-defensins 1–3 and cathelicidin), and are secreted by lung and trachea epithelia (cathelicidin) [3]–[6]. As a first line of innate defense, AMPs serve to limit bacterial colonization of mucosal surfaces [7]–[11]. Bacteria therefore adapt to resist AMP lethality through a series of countermeasures: remodeling the bacterial outer membrane surface to dampen charge and alter hydrophobicity [1], [12]–[14], export of AMPs via multiple transferable resistance (MTR)-mediated efflux pumps [15], secretion of exoproteases for AMP degradation [16], secretion of bacterial molecules to suppress host innate defense [17], [18], and release of proteins that function to adsorb extracellular AMPs [19].

Nontypeable *Haemophilus influenzae* (NTHI) is a commensal of the human nasopharnyx, yet causes opportunistic diseases such as conjunctivitis, sinusitis, exacerbations of chronic obstructive pulmonary disease, complications of cystic fibrosis and chronic and acute otitis media [20]–[25]. During the transition from a commensal to pathogen, NTHI must acquire nutrients and defend against host innate immune defense strategies including increased production of AMPs in response to infection. NTHI outer membrane remodeling provides a first line of defense against cationic AMPs. Lysenko and colleagues demonstrated that the presence of phosphorylcholine, a phase variable modification of NTHI lipooligosaccharide, alters outer membrane hydrophobicity that confers resistance to the cathelicidin LL-37 [26]. Additionally, HtrB is required for hexaacylation of NTHI lipid A, thus mutants lacking *htrB* are unable to fully acylate their lipid A rendering NTHI susceptible to AMP mediated killing [27].

NTHI lack the other described resistance mechanisms such as AMP efflux or exoprotease activity. The *sap* (sensitivity to antimicrobial peptides) operon encodes an inner membrane ABC-transporter, previously shown to play a crucial role in defense against AMPs [28]–[36]. Previously, we demonstrated that NTHI SapA, the periplasmic substrate binding protein of the Sap transporter, binds AMPs [37]. NTHI strains deficient in either SapA or the SapD ATPase are susceptible to killing by recombinant chinchilla β-defensin-1, an orthologue of human β-defensin-3, sharing 77% amino acid identity [37], [38]. Moreover, SapA and SapD are required for virulence in a mammalian host [37], [38]. Expression of the *sap* operon is up-regulated in vivo during NTHI-induced otitis media and in response to AMP exposure in vitro [37], [38]. The mechanism by which the Sap transporter complex confers AMP resistance remains unknown.

Here, we demonstrated that NTHI lacking the ligand binding protein SapA were directly susceptible to AMP exposure in the mammalian host, as neutralization of cBD-1 in vivo reversed attenuation and clearance of the *sapA* mutant strain. Further, we describe a novel mechanism of AMP import into the bacterial cytoplasm, which is dependent upon Sap transporter function. The Sap-permease was required for cytoplasmic localization and in a Sap-permease deletion strain, AMPs accumulated in the bacterial periplasm. Since AMPs were susceptible to intracytoplasmic peptidase activity, we hypothesize that AMP import, coupled with cytoplasmic degradation, decreases AMP accumulation in the bacterial periplasm and protects the bacterium from subsequent perturbation of the bacterial cytoplasmic membrane, a counter strategy to evade innate immune defense and ultimately benefit the bacterium nutritionally.

## Results

### SapA is required for NTHI to directly counter host defensin microbicidal activity *in vivo*

Previously, we demonstrated that the SapA periplasmic binding protein is required for NTHI to persist and cause disease in the middle ear of a mammalian model of otitis media [38]. In this co-infection model of middle ear disease (equal amounts of the wild type NTHI strain 86-028NP and the isogenic *sapA* mutant strain were introduced directly into the middle ear) we observed significant attenuation of the *sapA* mutant strain, which was unable to compete for survival and cleared from the middle ear. We subsequently demonstrated that recombinant SapA binds the AMP molecule, chinchilla β-defensin 1 (cBD-1) *in vitro* [37], and further, binds numerous AMPs, including the human β-defensin 3 (hBD-3) and cathelicidin (LL37) peptides [39]. These data support a mechanism whereby SapA confers protection from AMP lethality *in vivo* during disease progression. To directly assess this hypothesis, we sought to neutralize activity of cBD-1 *in vivo* and monitor the consequence of this treatment on survival of the SapA-deficient strain during a co-infection model of otitis media. We predicted that SapA was required by NTHI to counter the defensin bactericidal activity *in vivo*, and that neutralization of cBD-1 would reverse attenuation and clearance of the SapA-deficient strain in this environment.

Altered expression of AMPs can impact the ability of bacteria to colonize a host [11], [40]. We previously demonstrated that β-defensin expression controls NTHI bacterial colonization of the nasopharyngeal mucosal surface in a chinchilla model of NTHI-mediated disease [41]. Neutralization of available native cBD-1 via passive inhalation of affinity-purified antibody assessed the direct contribution of AMPs in mediating NTHI colonization levels. Here, we further developed and utilized this methodology to assess the direct contribution of AMPs in mediating attenuation of *sapA* mutant infection in the middle ear. We delivered affinity-purified anti-recombinant cBD-1 [(r)cBD-1] polyclonal antibody (or pre-immune serum as a negative control) to the chinchilla middle ear cavity (*n* = 5 per cohort) via the superior bullae, to inhibit the activity of native cBD-1. Twenty minutes after pre-treatment, we challenged chinchillas transbullarly with NTHI in a co-infection model, then measured the relative concentration of wild type and *sapA* mutant present in middle ear effusions over a 14 day period. A second dose of neutralizing antibody (or pre-immune serum) was delivered 24 hours after bacterial challenge.

**Figure 1:**
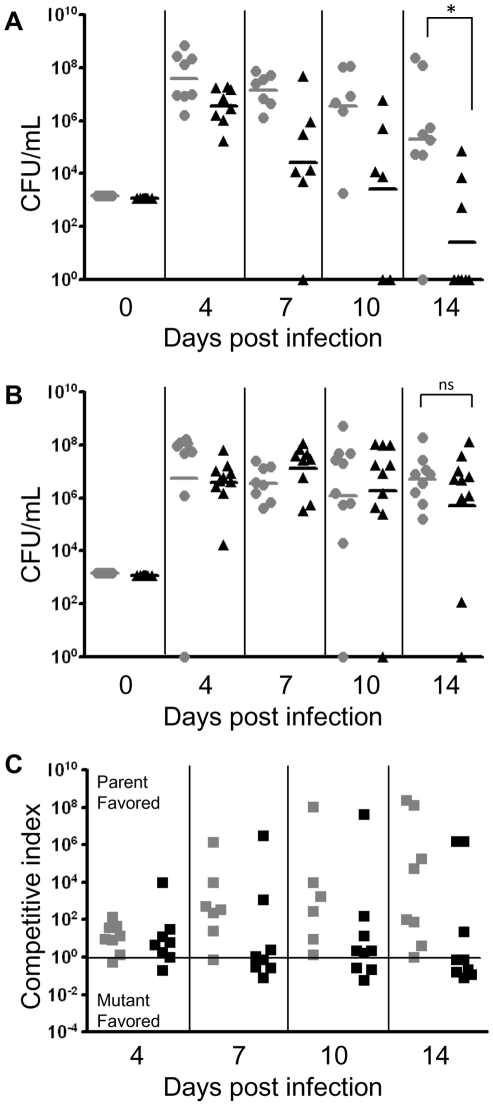
SapA is required for NTHI to directly counter host defensin lethality *in vivo.* Chinchilla middle ears were pre-treated with (r)cBD-1 antiserum (B) or pre-immune serum (A) then co-infected with a mixture of wild type NTHI strain 86-028NP and the isogenic *sapA* mutant to determine the consequence of neutralization of native cBD-1 on persistence of the *sapA* mutant in vivo. A second dose of serum was delivered one day after bacterial challenge. (A) The *sapA* mutant strain (black triangles) was unable to persist in the middle ear, and showed a significant decrease in colonization relative to that of the wild type strain (gray circles) 14 days after infection (p<0.01). (B) Neutralization of cBD-1 restored the ability of the *sapA* mutant strain to persist in the middle ear, at levels equal to or greater than that of the wild type strain. (C) Competitive index ratios (wild type to *sapA* mutant strain) were determined for each cohort receiving either the pre-immune (gray squares) or (r)cBD-1 (black squares) neutralizing antiserum. The black line indicates a competitive index of 1 (equal wild type to *sapA* mutant ratio). Black and gray horizontal lines (panels A and B) represent the geometric mean value of each cohort (n = 5 animals per cohort). Statistical significance was determined by non-parametric Mann-Whitney U test of geometric means, significance at p≤0.05.

We demonstrated that animals receiving anti-(r)cBD-1 neutralizing antibody remained colonized with the *sapA* mutant strain (Figure 1, panel B), which was sufficiently able to compete (in many cases out compete) with the wild type strain as shown in competitive index calculations (Figure 1, panel C). In contrast, the SapA-deficient strain is attenuated in animals receiving pre-immune serum (Figure 1, panel A). Consistent with our previous findings, we demonstrated that the *sapA* mutant was unable to persist over the course of infection (Figure 1, Panel A), and was significantly impaired (p<0.01) in its ability to infect the middle ear compared to that of the wild type strain 14 days after infection. Additionally, as previously described, anti-(r)cBD-1 antibody or the control pre-immune serum was not bactericidal against NTHI at concentrations used for AMP neutralization [41]. These data demonstrate that SapA functions to counter AMP lethality during NTHI infection in vivo. Since SapA binds and delivers periplasmic substrate to the inner membrane Sap transporter complex, we were interested to determine whether AMP molecules are transported to the cytoplasm of intact cells and whether this transport was dependent upon Sap permease activity.

### Co-fractionation of AMPs in cytoplasmic extracts of NTHI

**Figure 2:**
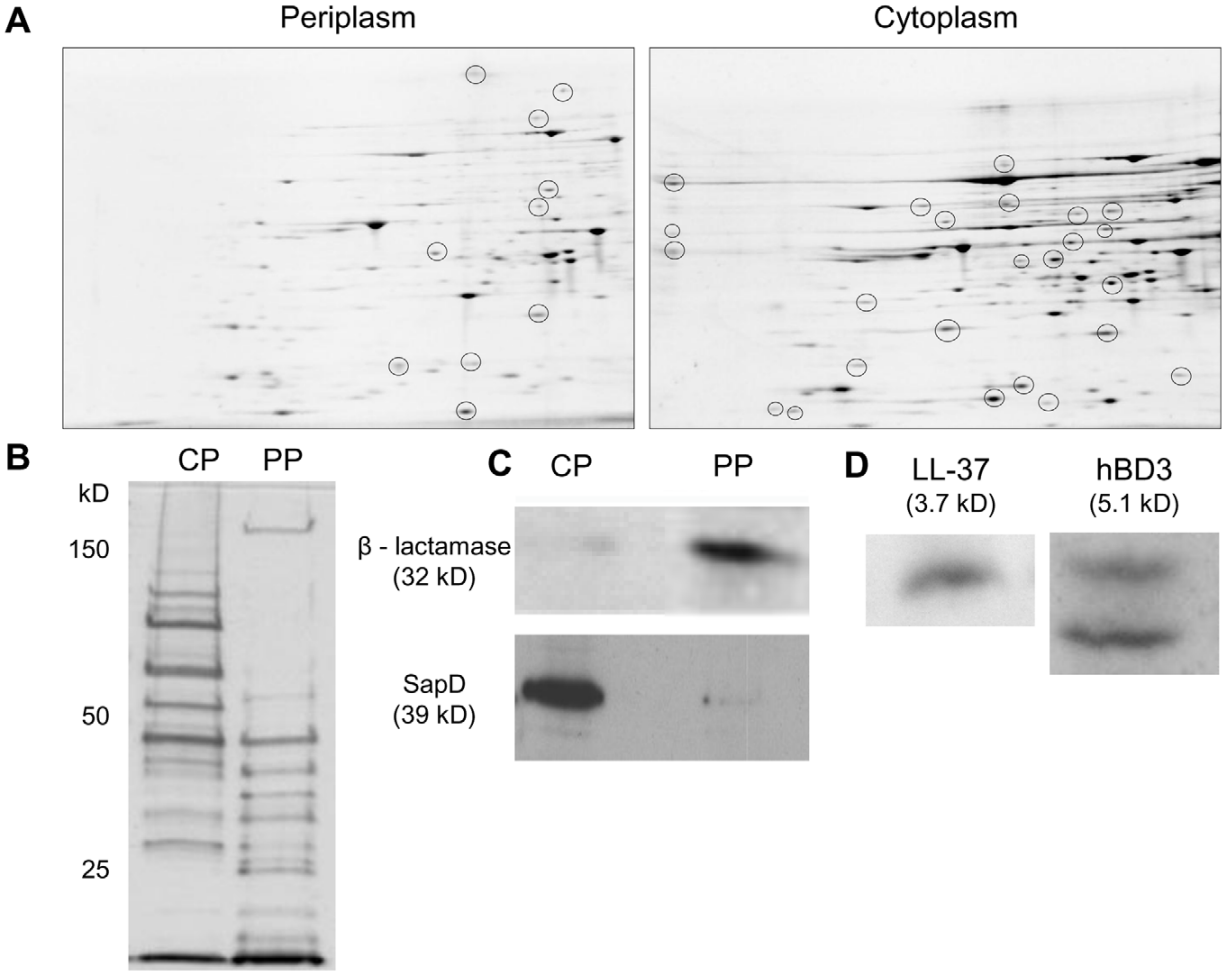
AMPs localize to cytoplasm-enriched fractions of NTHI. NTHI was fractionated to obtain cytoplasm and periplasm-enriched fractions. (A) Two dimensional gel electrophoresis of enriched fractions confirmed the presence of unique proteins in the periplasm and cytoplasm-enriched fractions. (B) Enriched fractions were separated by SDS-PAGE (12%) and silver stained to determine unique proteomic profiles. (C) Confirmation of bacterial fractionation by immunodetection of the periplasmic enzyme, β – lactamase and the cytoplasmic ATPase protein, SapD. (D) NTHI strain 86-028NP was exposed to LL-37 or hBD3 at sublethal levels for 30 minutes, fractionated and cytoplasm-enriched fractions were separated on a 16.5% Tris-Tricine gel and AMPs were detected by Western immunoblot analysis. No AMPs were detected in cytoplasm-enriched fractions prepared from cells alone.

We predicted that AMP substrates, bound by the SapA periplasmic binding protein, would be transported into the bacterial cytoplasm. Thus, we sought to obtain cytoplasm-enriched fractions of NTHI by bacterial fractionation and monitor the presence of AMPs in these fractions following exposure to sub-lethal concentrations of hBD3 or LL37. Traditional methods previously used to fractionate *Escherichia coli* were not successful with the prototypic NTHI clinical isolate strain, 86-028NP. Therefore, we utilized membrane destabilization and differential centrifugation (see Materials and Methods) and obtained both periplasm and cytoplasm-enriched fractions from lysed NTHI cells. The protein profiles of these two fractions were unique when visualized by two dimensional gel electrophoresis (Figure 2A) and SDS-PAGE analysis (Figure 2B). We confirmed fractionation by immunodetection of the periplasmic enzyme β-lactamase and the cytoplasmic protein SapD (Figure 2C). β-lactamase was localized to periplasm-enriched fractions and the cytoplasmic protein SapD was only detected in the cytoplasm-enriched fraction (not present in the periplasm). Importantly, we demonstrated that hBD-3 and LL37 co-fractionated with cytoplasm-enriched fractions of NTHI, demonstrating that AMPs, even at sublethal concentrations, gained access to the bacterial cytoplasm of intact NTHI (Figure 2D).

### AMP structural classes display different kinetics of transport into the bacterial cytoplasm

**Figure 3:**
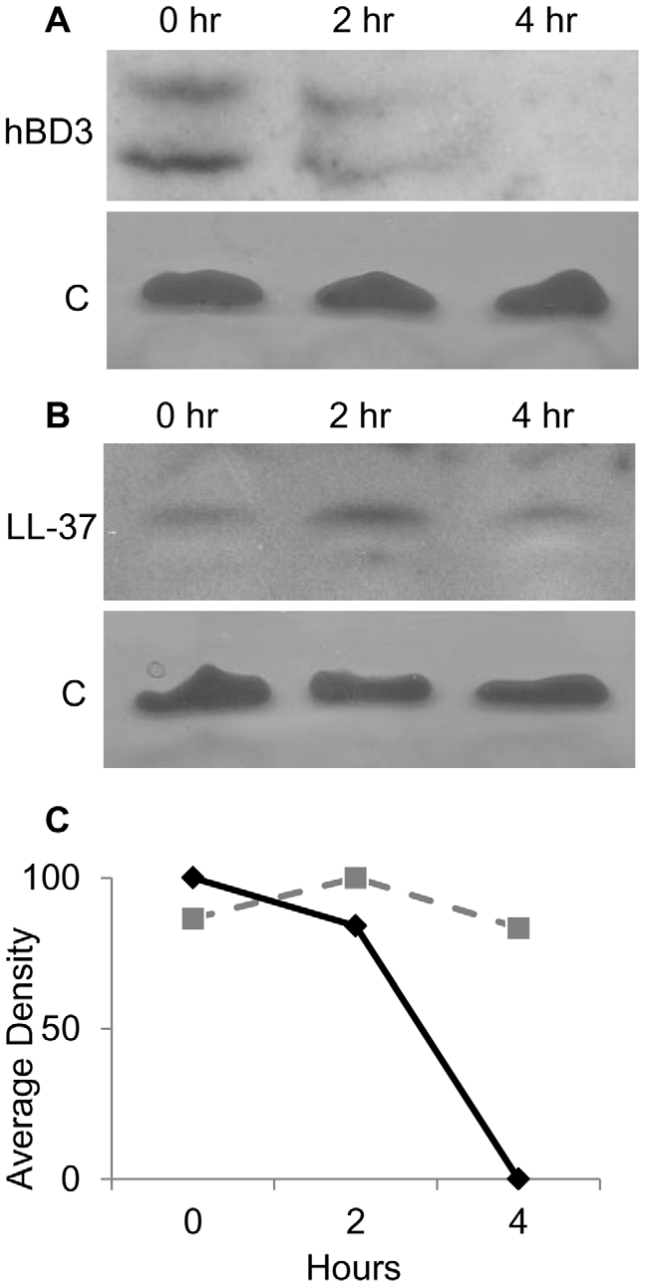
*In vivo* degradation of AMPs in NTHI cytoplasm. The parental NTHI strain was exposed to AMPs for 30 minutes, washed, resuspended in buffer without AMPs, and incubated for a chase period of 0, 2, or 4 hours followed by cell fractionation. Enriched cytoplasmic fractions were separated by SDS-PAGE on a 16.5% Tris-Tricine gel and hBD-3 (A) or LL-37 (B) was detected by immunoblot analysis. Samples were matched for viability, normalized for protein amount, and confirmed by silver stain of lipopeptide [C] contained in enriched cytoplasmic samples. Average density of bands is represented graphically (bottom panel C). There was no signal for control samples from cells incubated in the absence of AMPs. Data are representative of three independent assays.

We hypothesized that AMP substrates bound by SapA are delivered to the inner membrane Sap transporter complex for energy dependent transport into the bacterial cytoplasm. Indeed, we demonstrated that AMPs localized to the bacterial cytoplasm of intact cells within 30 minutes of exposure to sublethal AMP concentrations. Thus, we sought to characterize the kinetics of localization to the cytoplasm. NTHI were incubated with AMPs for 30 minutes (pulse period), cells were washed to removed unbound AMPs and then incubated in buffer alone (without AMPs) for 0, 2, or 4 hours (chase period). Incubation of AMP-exposed NTHI in buffer, during this chase period, did not affect NTHI viability during the time course (data not shown). At each time point, cytoplasm-enriched fractions were monitored for the presence of AMP molecules by immunoblot (Figure 3). We observed maximal import of hBD3 at the earliest time point tested (Figure 3A and 3C). Interestingly we observed a decrease in the detection of hBD3 over the remainder of the chase period (from 0 to 4 hours, Figure 3A and 3C). Import of hBD-3 was dependent upon SapA production as hBD-3 did not localize to the cytoplasm in a SapA-deficient strain (data not shown). Although similarly detected in the bacterial cytoplasm following the 30 minute pulse period (0 hr), LL-37 peptide appeared to accumulate during the first 2 hours of the chase period and decrease only slightly when incubated an additional 2 hours (4 hr, Figure 3A and 3C). These data suggest differential kinetics of AMP accumulation within NTHI, likely related to AMP structure and charge. We were unable to follow the fate of LL37 beyond the 4 hour chase period due to loss of NTHI viability.

### AMPs are degraded by cytoplasmic peptidase activity

**Figure 4:**
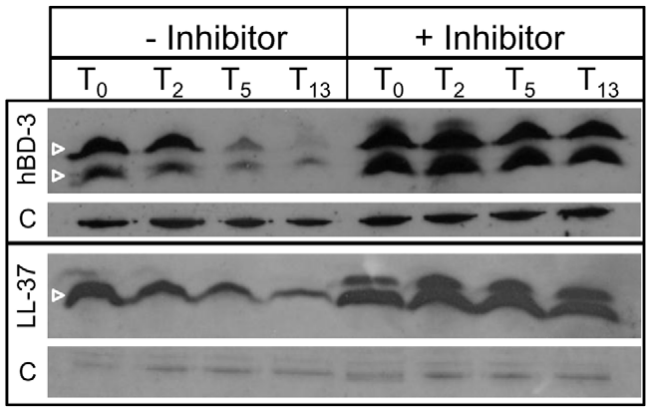
Degradation of AMPs by cytoplasmic peptidase activity. The parental NTHI strain was fractionated to obtain cytoplasm-enriched fractions. This fraction was combined with hBD-3 or LL37 in the presence or absence of a protease inhibitor cocktail (−/+ Inhibitor) and incubated for 0, 2, 5 or 13 hours. Samples were separated by SDS-PAGE on a 16.5% Tris-Tricine gel, and hBD3 or LL-37 was detected by immunoblot analysis. Samples were matched for viability and normalized for protein amount, as shown [C] for both hBD3 and LL-37.

The absence of known MTR in NTHI [15], combined with our observation of decreasing AMP levels in the cytoplasm, led us to hypothesize that the AMPs are degraded in the cytoplasm. To determine susceptibility of AMPs to peptidase activity, we incubated cytoplasm-enriched fractions of NTHI with hBD-3 or LL37 for 0, 2, 5, or 13 hours in the presence or absence of a protease inhibitor cocktail mix (−/+ inhibitor , Figure 4). At each time point, samples were collected and monitored for AMP by immunoblot (Figure 4). Similar to our observations in intact cells, we observed nearly complete loss of hBD-3 detection within 5 hours whereas LL37 loss was not observed to the same extent. After overnight incubation (T_13_) however, LL-37 detection was dramatically reduced. In both cases, AMPs were susceptible to cytoplasmic fraction peptidase activity that was blocked in the presence of protease inhibitors (Figure 4, + inhibitor). Collectively, these data suggest that AMP molecules are susceptible to cytoplasmic protease activity, and are likely beneficial to NTHI for metabolic purposes.

### Transport of antimicrobial peptides to the cytoplasm of NTHI requires the Sap permease

**Figure 5:**
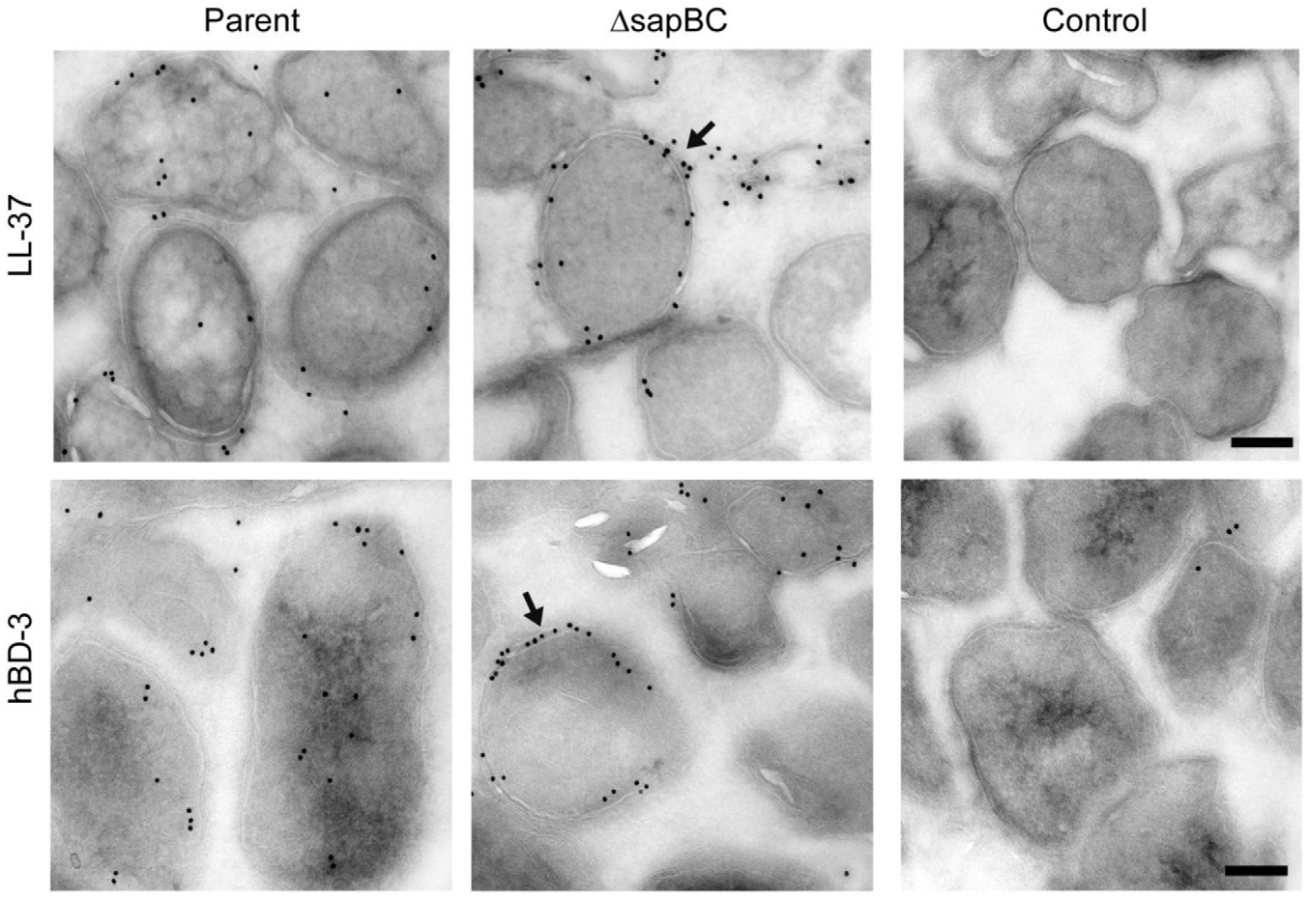
The Sap transporter inner membrane permease is required for AMP transport to the bacterial cytoplasm. Parent and permease-deficient NTHI were incubated with sublethal concentrations of LL-37 or hBD3 for 30 minutes, washed, fixed, sectioned (50 nm) and immunogold labeled for AMP as visualized by transmission electron microscopy (TEM) analysis. Parallel samples not exposed to AMPs yet processed in parallel served as a labeling control. Scale bar = 200nm.

The Sap transporter belongs to a family of ABC transporters that recognize and transport substrates that are typically small and cationic in nature [29]. We previously demonstrated that AMPs can displace additional substrates previously bound by SapA, and that mutations in Sap transporter proteins alter NTHI susceptibility to AMP exposure [37]–[39]. We hypothesized that the Sap transporter may mediate the transfer of AMPs across the cytoplasmic membrane, as a mechanism of AMP transport into the cell. We therefore generated a deletion mutant lacking the permease components of the Sap transporter (SapB and SapC) and determined the subcellular localization of LL-37 and hBD3. NTHI (parent and *sapBC* permease mutant) were exposed to sub-lethal concentrations of either LL-37 or hBD3 for 30 minutes and processed to assess AMP localization by transmission electron microscopy, to visualize AMP localization in intact cells. We observed that both LL-37 (Figure 5, top panels) and hBD3 (Figure 5, bottom panels) localized to the periplasm and the cytoplasm of the parental cells. Accumulation of AMPs was not observed in either the bacterial outer or inner membranes under these conditions. In contrast, neither LL-37 nor hBD3 were observed in the cytoplasm of the *sapBC* permease-deficient cells, yet a striking accumulation of AMPs was observed in the periplasm and cytoplasmic membrane. These data suggest that subcellular localization of AMP molecules to the bacterial cytoplasm is dependent upon Sap-mediated transport. Consistent with this observation, *sapBC* permease-deficient cells are more sensitive to AMP exposure (data not shown). This targeted transport of AMPs, to the cytoplasm for degradation, may serve to reduce periplasmic accumulation of AMPs, and thus protect the cytoplasmic membrane from AMP association and lethality. Thus, Sap-mediated transport of AMP to the cytoplasm for degradation serves as a newly described subversion mechanism that would provide a nutritional benefit to the bacterium thus coupling innate immune resistance to metabolic activity.

## Discussion

The ability of NTHI to persist in both the commensal and pathogenic lifestyles requires mechanisms to avert the host innate immune response. The epithelium in the upper airway produces AMPs that assist in the control of NTHI [41]–[43]. This led us to investigate the mechanisms used by NTHI to evade killing by AMPs. Modifications of the outer membrane primarily serve as a first line of defense against AMP lethality including addition of phosphorylcholine to lipooligosaccharide to repel positively charged AMPs [26] and lipid A acylation to increase NTHI hydrophobicity, thus altering membrane permeability [27]. Although effective as a first line defense strategy, AMP accumulation and disruption of the outer membrane, periplasmic localization and permeabilization of the cytoplasmic membrane is microbicidal. Many bacteria possess additional mechanisms to detect and resist AMP insult shown to be regulated through two-component regulatory signaling [44], [45], release of extracellular peptidases [16], secreted proteins to bind AMPs [19], and AMP efflux pumps [15], [46], [47]. NTHI however, lacks two-component regulatory systems that sense AMP exposure, and additional mechanisms of AMP resistance have not been identified.

**Figure 6:**
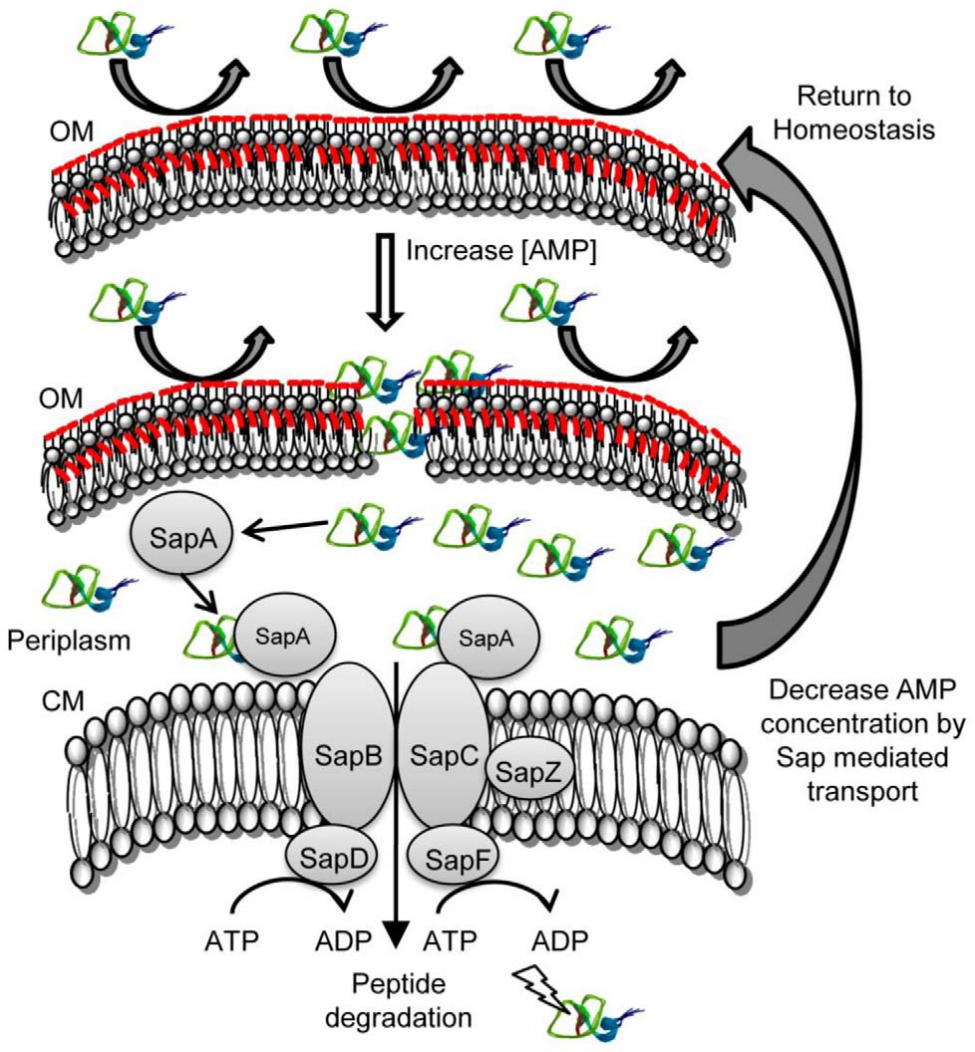
Model of Sap-dependent import of AMP molecules and subsequent degradation. In the presence of low concentrations of AMP, NTHI are able to resist lethality by modification of the outer membrane (OM; ChoP and Lipid A acylation, red) and subsequent repulsion of cationic charged peptides away from the bacterial cell surface. An increase in local concentrations of AMP increases production of the Sap transporter which functions to bind and transport periplasmic AMPs across the NTHI cytoplasmic membrane (CM). Transported AMPs are susceptible to proteolytic degradation. A reduction in the critical threshold concentration of AMPs in the periplasm returns NTHI to a homestatic state of innate immune resistance.

We previously demonstrated that the Sap transporter conferred NTHI resistance to AMPs. The periplasmic binding protein, SapA, binds AMPs [37], contributes to AMP resistance [38] and is also upregulated in response to AMP exposure during otitis media. This, taken with our data presented here, suggests that resistance to AMP lethality, conferred via Sap mediated transport of AMPs, is important for NTHI pathogenesis. Since we observed marked attenuation of Sap transporter mutants in vivo [37], [38], we predicted that neutralization of AMPs would rescue this attenuated phenotype, supporting a critical role for Sap-dependent AMP import in NTHI pathogenesis. In support of this, in vivo neutralization of the hBD3 orthologue, chinchilla β-defensin-1, which is highly expressed in the chinchilla upper respiratory tract [48], results in increased colonization by NTHI [41]. These data suggest that AMPs play an important role in limiting infection in a mammalian host. Indeed, alterations in AMP production can affect the ability of other microorganisms to colonize a host [11], [40], [49]. The ability of NTHI to exploit mechanisms to resist AMP lethality may equip transition to that of opportunistic pathogen and ability to cause disease. We demonstrated here that neutralization of β-defensin activity in the middle ear of a mammalian model of otitis media rescues the attenuated phenotype of the SapA-deficient strain, such that the mutant strain is no longer cleared from this environment (Figure 1). This is the first direct evidence that SapA contributes to bacterial virulence by conferring protection from AMP lethality in vivo. Having established this, we monitored the consequence of SapA-AMP interaction in intact bacterial cells. We describe a novel mechanism of inner membrane transport of AMP molecules, via the Sap ABC transporter, to the bacterial cytoplasm resulting in AMP degradation (Figure 6). This direct mechanism of AMP influx serves to decrease both periplasmic and inner membrane accumulation of AMPs and protect NTHI from AMP bactericidal effects. Current models predict that AMP-induced microbicidal activity results from transmembrane pore formation subsequent to AMP accumulation at the cell surface [1]. However, we observed by TEM analysis, that AMPs localized to the bacterial cytoplasm, even when NTHI were exposed to sublethal concentrations that do not appear to accumulate in the bacterial membranes, suggesting a mechanism whereby bacteria can directly import AMP molecules. Since accumulation of AMPs in the bacterial cytoplasm would likely be detrimental, we hypothesized that AMPs are subsequently targeted for cytoplasmic degradation. Indeed, we observed that hBD3, localized to the bacterial cytoplasm, was susceptible to cytoplasmic degradation, as the ability to detect hBD3 decreased over time (Figure 3A, C). Although we did not observe an appreciable decrease in LL-37 detection following uptake into intact cells, we demonstrated that LL37, like hBD-3, was susceptible to peptidase activity in cytoplasm-enriched fractions (Figure 4). These data suggest differential kinetics of uptake and degradation, likely dependent upon AMP structure and charge. In support of differential kinetic uptake of AMP molecules, we previously demonstrated that LL-37 and hBD3 differ in their abilities to bind and displace the iron-containing compound heme, also shown to bind SapA [39]. Consistent with these studies, cytoplasmic accumulation of LL-37 appeared delayed, relative to hBD3 (Figure 3C). Although we have not, as of yet, identified the binding sites for these SapA ligands, our data indicate that hBD3, which is highly cationic, may preferentially bind SapA based upon charge (unpublished). Additionally, it has been shown that the cathelicidin LL-37 contains proline-rich sequences that resist degradation by serine proteases [50] and NTHI lack PgtE and OmpT homologues, shown to mediate degradation of alpha-helical peptides [51]–[54], which may explain our observation that LL-37 appears more resistant to proteolysis within the bacterial cytoplasm. NTHI encodes homologues of bacterial cytoplasmic proteases, ClpX and Lon proteases, shown to contribute to AMP resistance in other microorganisms [55], [56]. The contribution of these proteases to AMP degradation in NTHI is currently unknown, but remains the focus of future work.

An alternative hypothesis is that an MTR drug efflux pump functions to export AMPs that have accumulated in the bacterial inner membrane and in the cytoplasm. It was recently shown that the MtrC periplasmic membrane fusion protein conferred resistance to LL-37 and β-defensins in *Haemophilus ducreyi* [47]. Interestingly, the *H. ducreyi* Sap transporter does not mediate β-defensin resistance [36], [47]. Although NTHI strain 86-028NP contains week homologs of the MTR system, our evidence suggests that this system is not functional or does not function to confer resistance to defensin or cathelicidin peptides, a well described function of the NTHI Sap transporter [37]–[39]. Further, we demonstrated that in vivo neutralization of native cBD-1 was sufficient to rescue competitive growth of the *sapA* mutant, suggesting that SapA was sufficient to mediate resistance to this defensin molecule in the host. Finally, a functional MTR system would be expected to export AMP molecules thus avoiding periplasmic, membrane and cytoplasmic accumulation of AMPs. We observed, in the absence of a functional Sap permease complex, AMP accumulation (Figure 5) and increased sensitivity to AMP-mediated killing (data not shown). We would expect, based upon these published reports that a functional MTR system would not result in this phenotype.

Development of antibiotic resistant strains secondary to conventional antibiotic use has prompted interest in the use of AMPs as therapeutic alternatives to treat bacterial infections [57]. The use of AMPs in therapeutic regimens may be hindered due to bacterial subversion mechanisms of AMP lethality [54]. Since the Sap transporter functions to protect against the accumulation of AMPs in the bacterial periplasm, and subsequent interaction with the cytoplasmic membrane, a mechanism to block transporter function may provide novel therapeutic supplements for NTHI infections. Delivery of small molecule inhibitors to block substrate binding and transport would confound an important AMP resistance mechanism in NTHI, rendering NTHI susceptible to innate immune attack while preserving the normal flora that are often disrupted by conventional antibiotic use.

The described novel mechanism of AMP import and subsequent degradation expands our understanding of host-pathogen interactions, particularly those that mediate resistance to key components of the host innate immune response. Future studies to identify AMP specific intracellular peptidases, AMP degradation products, and metabolic consequences of targeted peptide degradation, are needed to better understand bacterial survival strategies in the hostile host environment. Further, a better understanding of bacterial mechanisms used to transition from commensal to that of an opportunistic pathogen will better equip the design of therapeutics to combat disease.

## Materials and Methods

### Ethics statement

All animal experiments were carried out in strict accordance with the accredited conditions in the Guide for the Care and Use of Laboratory Animals of the National Institutes of Health. The protocol was approved by the Institutional Animal Care and Use Committee (Welfare Assurance Number A3544-01) at The Research Institute at Nationwide Children's Hospital, AR08-00027.

All experimental procedures were performed under xylazine and ketamine anesthesia, and all efforts were made to minimize suffering.

### Animals

Healthy adult chinchillas (*Chinchilla lanigera*), purchased from Rauscher's Chinchilla Ranch (LaRue, OH), were used for these studies, after allowing them to acclimate to the vivarium for 7 to 10 days. Chinchillas were anesthetized with xylazine (2 mg/kg, Fort Dodge Animal Health, Fort Dodge, IA) and ketamine (10 mg/kg), Phoenix Scientific Inc., St. Joseph, MO), and nasopharyngeal lavage fluids were obtained by passive inhalation of 500 µl of pyrogen-free sterile saline into one naris with recovery of lavage fluid from the contralateral naris as liquid was exhaled. Middle ear fluids were recovered via epitympanic tap through the superior bullae, and directly obtained from the inferior bullae behind the tympanic membrane. All animal experiments were performed using accredited conditions for animal welfare approved by the Institutional Animal Care and Use Committee (Welfare Assurance Number A3544-01) at The Research Institute at Nationwide Children's Hospital, AR08-00027.

### Bacterial strains and culture conditions

Nontypeable *Haemophilus influenzae* strain 86-028NP is a minimally passaged clinical isolate obtained at Nationwide Children's Hospital in Columbus, Ohio. This prototypic wild type strain has been sequenced and extensively characterized in chinchilla models of otitis media [58], [59]. The parental NTHI strain 86-028NP:: *rpsL* is a streptomycin resistant strain constructed as previously described [60]. The *sapBC* permease deletion mutant was constructed as an unmarked, non-polar deletion mutation of the *sapB* and *sapC* genes by recombineering strategy as previously described [39]. Bacterial strains were grown on chocolate II agar (Becton Dickinson, Sparks, MD) or in brain heart infusion broth supplemented with 2 µg heme/mL and 1 µg NAD/mL (sBHI). Bacteria were cultured from overnight growth on chocolate II agar, resuspended in sBHI to OD_490_ = 0.65 (equivalent to 1×10^8^ CFU/ml), diluted 1:5 into fresh sBHI medium and grown to mid-log phase (3 hours) at 37°C, 5% CO_2_, static.

### Antibody-induced neutralization of native cBD-1 in vivo

In vivo neutralization of chinchilla β-defensin 1 (cBD-1) was performed as previously described with the following modifications [41]. We prepared and purified (r)cBD-1 based upon previously published methods [41], [48]. A HiTrap protein G HP column (GE healthcare, Pittsburgh, PA) was used to affinity purify total IgG from rabbit anti-(r)cBD-1 and the cognate pre-immune serum. One milliliter of serum was dialyzed [3.5 kDa molecular weight cutoff (MWCO), EMD Chemicals Inc., San Diego, CA] at 4°C against 20 mM sodium phosphate buffer, pH 7.0 and applied to the affinity column. Immunoglobulins were eluted from the affinity matrix with 0.1 M glycine-HCl, pH 2.7 in one milliliter fractions into eppendorf tubes that contained 200 µl of 1.0 M Tris-HCl, pH 9.0, to neutralize the acidic elution conditions. Samples that contained the greatest amount of protein (fractions 1 and 2) were pooled and dialyzed overnight at 4°C against sterile saline. Protein concentrations of the anti-(r)cBD-1 and the pre-immune serum were determined using Coomassie Plus Protein Assay Reagent (Thermo Scientific, Rockford, IL). We confirmed that anti-(r)cBD-1 antiserum, and not the pre-immune antiserum, bound purified (r)cBD-1 by immunoblot (data not shown). Five adult chinchillas were administered 90 µg anti-cBD-1 or pre-immune rabbit immunoglobulin, in a total volume of 200 µl. Antisera were administered by passive inhalation of droplets of the solution delivered to the nares or direct transbullar inoculation of the middle ears of anesthetized chinchillas. Animals were then placed in a prone position for 20 minutes prior to intranasal challenge with approximately 5.0×10^7^ cfu NTHI wild type strain 86-028NP mixed with 5.0×10^7^ cfu *sapA* mutant (co infection) in a 100 µl volume or transbullarly with 2.5×10^3^ cfu NTHI wild type strain 86-028NP mixed with 2.5×10^3^ cfu *sapA* mutant in a 200 µl volume. Nasopharyngeal lavage and epitympanic taps were performed 1, 2, 4, 7, 10, 14 days after NTHI co-challenge, and bacterial counts were determined by dilution plating of bacteria on chocolate agar.

### NTHI fractionation

Parental NTHI strain 86-028NP::*rpsL* was grown to mid-log phase in brain heart infusion broth supplemented with 2 µg heme/ml and 2 µg NAD/ml (sBHI). Cells were pelleted by centrifugation and resuspended in 1X phosphate buffered saline containing 2 mg Polymyxin B sulfate (Sigma-Aldrich, St. Louis, MO)/ml, 0.05% glycerol, to bind lipooligosacharide and generate spheroplasts, thereby releasing periplasmic contents to the supernatant. Following incubation for 1 hour at 37°C on a rotating platform (100 rpm), spheroplasts were pelleted by centrifugation and resuspended in 1 ml 10 mM HEPES pH 7.4 (Sigma-Aldrich, St. Louis, MO), 0.05% glycerol and subsequently lysed by freeze-thaw method (10 cycles, −78°C to 37°C). Lysed spheroplasts were then incubated at room temperature, on an orbital rocker, with 1 µl Benzonase Nuclease (Novagen, Darmstadt, Germany) and 5 µl 1 M MgCl_2_ (Ambicon, Alamo, CA)) to a final concentration of 5 µM. Bacterial membranes were pelleted by ultracentrifugation (40,000 rpm for 1 hour, 4°C) and cytoplasmic proteins were removed in the supernatant. Cytoplasm and periplasm-enriched fractions were observed by two dimensional gel electrophoresis (Bio-Rad, Hercules, CA) of enriched fractions, stained with SYPRO Ruby Protein Gel Stain (Invitrogen, Carlsbad, CA) and imaged at 535 nm (Kodak Image Station 2000, New York, NY). To ensure successful NTHI fractionation, cytoplasm and periplasm-enriched fractions were analyzed by silver stain (Pierce, Rockford, IL) following SDS-PAGE separation. In addition, NTHI were exposed to a sub-lethal concentration of 15 µg ampicillin/mL to induce expression and trafficking of β-lactamase to the NTHI periplasm. Periplasm and cytoplasm-enriched fractions were separated by SDS-PAGE and transferred to nitrocellulose (Bio-Rad, Hercules, CA). Membranes were then blocked in 3% nonfat dried milk and probed for the periplasmic protein, β-lactamase, by incubating with mouse anti- β-lactamase for 45 minutes or the cytoplasmic protein, SapD, by incubating with rabbit anti-SapD overnight. Membranes were then washed, incubated with goat anti-mouse IgG (H+L) HRP conjugate (Invitrogen, Carlsbad, CA) for 30 minutes or goat anti-rabbit IgG (H+L) HRP conjugate (Invitrogen, Carlsbad, CA) for 1 hour, washed, and peroxidase activity was detected using Amersham ECL Western Blotting Detection Reagents (GE Healthcare, Little Chalfont, Buckinghamshire, UK).

### In vivo pulse-chase analysis to determine AMP localization

Parental NTHI strain 86-028NP::*rpsL* were grown to mid-log phase in sBHI, normalized for cell number, and pelleted. Pellets were resuspended in 10 mM sodium phosphate buffer pH 7.4, supplemented with 2% sBHI. Cultures were incubated with sublethal concentrations of LL-37 or hBD3 as previously described, for a pulse period of 30 minutes. Cells were then pelleted by centrifugation, washed, and resuspended in 10 mM sodium phosphate buffer pH 7.4, supplemented with 2% sBHI. Cultures were incubated at 37°C, 5% CO_2_, static for a chase period of 0, 2, or 4 hours. Samples were transfered to ice and fractionated to obtain periplasm and cytoplasm-enriched fractions as described above. Enriched fractions were lyophilized (Labconco, Kansas City, MO) overnight and subsequently resuspended in 10 mM HEPES pH 7.4, 0.05% glycerol, then normalized for equal amounts of protein using Coomassie Plus Protein Assay Reagent (Thermo Scientific, Rockford, IL) prior to separation by SDS-PAGE (16.5% Ready Gel Tris-Tricine Precast Gels, Bio-Rad, Hercules, CA). Equal detection of NTHI lipopetide [61] by silver stain (Pierce, Rockford, IL) confirmed normalization of samples. Samples containing LL-37 or hBD3 were transferred to PVDF (Bio-Rad, Hercules, CA) or nitrocellulose (Bio-Rad, Hercules, CA), and blocked in 3% skim milk or 5% BSA, respectively. Membranes were incubated with rabbit anti-LL-37 (Phoenix Pharmaceuticals, Burlingame, CA) or goat anti-hBD3 (Leinco Technologies, St. Louis, MO) overnight at 4°C, washed, and incubated with goat anti-rabbit IgG (H+L) HRP conjugate (Invitrogen, Carlsbad, CA) or rabbit anti-goat IgG (H+L) HRP conjugate (Invitrogen, Carlsbad, CA), respectively. Membranes were washed and peroxidase activity was detected using SuperSignal West Femto Chemiluminescent Substrate (Thermo Scientific, Rockford, IL).

### Degradation of antimicrobial peptides in cytoplasm-enriched fractions

NTHI cells were fractionated as described above. Cytoplasm-enriched fractions were concentrated in Amicon centrifuge filters (3.0kDa MWCO) and normalized for equal amounts of protein using Coomassie Plus Protein Assay Reagent (Thermo Scientific, Rockford, IL). Cytoplasmic peptidase activity was monitored by incubating 8 µg of cytoplasm-enriched fractions with LL37 (40 ng) or hBD-3 (20 ng) in siliconized glass vials and incubated at 37°C for 2, 5.5 and 13 hours. In parallel, a protease inhibitor cocktail set was included (Calbiochem, LaJolla, CA). Following incubation, samples were separated on a SDS-PAGE gel (16.5% Ready Gel Tris-Tricine Precast Gels, Bio-Rad, Hercules, CA). Samples containing LL-37 or hBD3 were transferred to PVDF (Bio-Rad, Hercules, CA) or nitrocellulose (Bio-Rad, Hercules, CA), and blocked in 3% skim milk or 5% BSA, respectively. Membranes were incubated with rabbit anti-LL-37 (Phoenix Pharmaceuticals, Burlingame, CA) or goat anti-hBD3 (Leinco Technologies, St. Louis, MO) overnight at 4°C, washed, and incubated with goat anti-rabbit IgG (H+L) HRP conjugate (Invitrogen, Carlsbad, CA) or rabbit anti-goat IgG (H+L) HRP conjugate (Invitrogen, Carlsbad, CA), respectively. Membranes were washed and peroxidase activity was detected using SuperSignal West Femto Chemiluminescent Substrate (Thermo Scientific, Rockford, IL).

### Cryo Immunoelectron microscopy

Parental strains NTHI 86-028NP::*rpsL* and the isogenic *sapBC* mutant strain were grown to mid-log phase in sBHI, normalized for cell number, and pelleted. Pellets were resuspended in 10 mM sodium phosphate buffer pH 7.4, supplemented with 2% sBHI. Human LL-37 cathelicidin (Phoenix Pharmaceuticals, Burlingame, CA) or human beta defensin 3 (PeproTech, Rocky Hill, NJ) were added to cultures at a final concentration of 0.25 µg LL-37/ml or 1 µg hBD3/ml. Cultures containing AMPs or cells alone were incubated at 37°C, 5% CO_2_, static for 30 minutes. Samples were transfered to ice and cells were pelleted by centrifugation, washed in 100 mM PIPES pH 7.0, and then resuspended in 100 mM PIPES. For immunolocalization of AMPs at the ultrastructural level, bacteria were fixed in 4% paraformaldehyde/0.05% glutaraldehyde (Polysciences Inc., Warrington, PA) in 100 mM PIPES/0.5 mM MgCl_2_, pH 7.2 (or PBS) for 1 hr at 4°C. Samples were then embedded in 10% gelatin and infiltrated overnight with 2.3 M sucrose/20% polyvinyl pyrrolidone in PIPES/MgCl_2_ at 4°C. Samples were trimmed, frozen in liquid nitrogen, and sectioned with a Leica Ultracut UCT cryo-ultramicrotome (Leica Microsystems Inc., Bannockburn, IL). 50 nm sections were blocked with 5% FBS/5% NGS for 30 min and subsequently incubated with rabbit anti-hBD3 (Leinco Technologies, Inc.) or rabbit anti-CAP-18 (LL-37) antibody for 1 hr at room temperature. Sections were then washed in block buffer and probed with 18 nm colloidal gold-conjugated anti-rabbit IgG (H+L) (Jackson ImmunoResearch Laboratories, Inc., West Grove, PA) for 1 hr at room temperature. Sections were washed in PIPES buffer followed by a water rinse, and stained with 0.3% uranyl acetate/2% methyl cellulose. Samples were viewed with a JEOL 1200EX transmission electron microscope (JEOL USA Inc., Peabody, MA). All labeling experiments were conducted in parallel with controls omitting the primary antibody. These controls were consistently negative at the concentration of colloidal gold conjugated secondary antibodies used in these studies.

### Accession numbers

Proteins discussed in this manuscript are listed followed by their corresponding UniProtKB (Universal Protein Knowledgebase) number. These included: SapA (Q4QL73_HAE18), SapB (Q4QL74_HAE18), SapC (Q4QL75_HAE18), SapD (Q4QL76_HAE18), SapF (Q4QL77_HAE18), OmpT (OMPT_ECOLI), PgtE (PGTE_SALTY), ClpX (Q4QMK4_ HAE18), Lon (Q4QN81_ HAE18), HtrB (Q4QKN9_ HAE18), β-Lactamase (A720C2_ HAE18).
